# Prevalence and Risk Factors Associated With Gestational Diabetes Mellitus Among Pregnant Women: A Cross-Sectional Study in Ghana

**DOI:** 10.3389/fcdhc.2022.854332

**Published:** 2022-03-22

**Authors:** Wina Ivy Ofori Boadu, Philomina Kugblenu, Ebenezer Senu, Stephen Opoku, Enoch Odame Anto

**Affiliations:** ^1^ Department of Medical Diagnostics, Faculty of Allied Health Sciences, College of Health Science, Kwame Nkrumah University of Science and Technology, Kumasi, Ghana; ^2^ Department of Molecular Medicine, School of Medicine and Dentistry, College of Health Science, Kwame Nkrumah University of Science and Technology, Kumasi, Ghana; ^3^ School of Medical and Health Sciences, Centre for Precision Health, Edith Cowan University, Perth, WA, Australia

**Keywords:** gestational diabetes mellitus, pregnant women, risk factors, prevalence, dietary lifestyle modification

## Abstract

Gestational diabetes mellitus (GDM) is a global public health issue that have serious consequences on mother and her child’s health. However, limited data is available on the prevalence of GDM and its associated risk factors in Ghana. This study investigated the prevalence and associated risk factors of GDM among women attending selected antenatal clinics in Kumasi, Ghana. This cross-sectional study included 200 pregnant women who attended antenatal clinics from Three-selected health facilities in the Ashanti Region, Ghana. Women already diagnosed of GDM were identified through their medical records and were confirmed based on the criteria of the International Association of Diabetes and Pregnancy Study Groups (IADPSG), which uses a fasting blood glucose of ≥ 5.1 mmol/L. A well-structured questionnaire was used to collect data on socio-demographic, obstetric, clinical and lifestyle risk factors. Multivariate logistic regression models were used to determine the independent risk factors of GDM. The overall prevalence of GDM among study participants was 8.5%. GDM was prevalent among age 26 and 30 years (41.2%), married participants (94.1%) with basic education (41.2%) and being Akan by ethnicity (52.9%). Previous history of oral contraceptive use (aOR: 13.05; 95% CI: 1.43–119.23, p=0.023), previous history of preeclampsia (aOR: 19.30; 95% CI: 2.15-71.63; p=0.013) and intake of soda drinks (aOR: 10.05, 95% CI: 1.19–84.73, p=0.034) were found to be independent risk factors of GDM. The prevalence of GDM was found to be 8.5% and this was associated with the previous use of oral contraceptives, history of preeclampsia and intake of soda drinks. Public health education and dietary lifestyle modification may be required for pregnant women who are at risk of GDM.

## Introduction

Diabetes in pregnancy affects women in one of two ways: pregestational (which includes type 1 and type 2 diabetes) or gestational diabetes mellitus (GDM). By the 1950s, the word “gestational diabetes” had been coined to describe what was believed to be a temporary disease that had a negative impact on foetal outcomes and then disappeared after birth (1).

GDM, is carbohydrate resistance that starts or is first recognized throughout pregnancy (2). This diagnosis does not extend to pregnant women who have already been diagnosed with diabetes before becoming pregnant. Gestational diabetes has a pathophysiology which is similar to that of type 2 diabetes. As the pregnancy progresses, tissue resistance to insulin increases, necessitating the use of more insulin (3). The demand is easily met in the vast majority of pregnancies, so the balance between insulin resistance and insulin supply is maintained. Women, on the other hand, become hyperglycaemic if resistance becomes dominant. This typically happens in the second trimester of pregnancy, with insulin resistance steadily increasing before delivery, and usually disappears quickly after delivery (4). In 2017, one in every seven births was diagnosed with GDM, according to the International Diabetes Federation (IDF) (5). Globally, the prevalence of GDM is estimated to be about 15%, according to a systemic review (6). GDM prevalence ranges from 2-6 percent in most racial/ethnic groups studied to, 10-20 percent in high-risk populations, with a growing trend across most racial/ethnic groups (7). Since ethnicity has such a strong impact on GDM, the prevalence rate varies by race. The prevalence of GDM in Sub-Saharan Africa is around 14% (8). As at 2014 in Ghana, GDM was discovered in 10% of pregnant women (9). The prevalence of gestational diabetes in Ghana is increasing at a very fast rate. In 2004 and 2015, studies conducted in Ghana indicated a prevalence of 0.5% and 9.3% respectively (10, 11).

The prevalence of GDM is projected to continue to increase due to the rising obesity epidemic, delayed childbearing, and multiple pregnancies (12). Gestational diabetes is known to have serious short and long-term effect on both the mother and foetus. GDM may cause pregnancy complications such as high blood pressure, heavy birth weight babies, and obstructed labour in the short term (11). GDM tends to be a major risk factor for the development of type 2 diabetes (T2D) and cardiovascular diseases in women (13–15). Obesity and T2D are more likely to occur in children whose mothers had diabetes during pregnancy (16).

The development of gestational diabetes has been associated with several predisposing factors. These predisposing factors can be studied under lifestyle, obstetric, socio-demographic and clinical risk factors. Some of the obstetric risk factors associated with GDM includes previous abortion, parity and stillbirth (17). Socio-demographic risk factors studied so far also include age, ethnicity and family history of diabetes (18, 19). Clinical risk factors assessed are obesity and hypertension. Lifestyle risk factors like diet, physical activity, smoking and alcoholism are also known to have a link with GDM (19).

Despite the fact that urine dipstick has been accepted as the major screening criteria for GDM in Ghana, it is limited to renal threshold, hence most pregnant women who develop GDM later in their pregnancy are still excluded during regular screening programs for women. The risk factors of gestational diabetes can be used as a better diagnostic tool for early screening of women at risk hence it is necessary to identify the socio-demographic, obstetric, clinical and lifestyle risk factors of GDM. However, in Ghana and especially in greater Kumasi metropolis, there is no enough data on the risk factors associated with GDM. Therefore, this study is warranted.

## Materials and Methods

### Study Design and Settings

This study is a hospital-based multi-centre cross-sectional study, conducted at the Antenatal Clinics of Kwame Nkrumah University of Science and Technology Hospital, Kumasi South Hospital and Saint Michael’s Hospital all in Kumasi Ghana, from April to July 2021.

### Study Population and Selection

A simple randomised sampling method was used to recruit a total of 200 pregnant women aged 16-45 years with gestational period between 16 and 40 weeks who were attending regular antenatal clinic at three selected hospitals in the Ashanti Region of Ghana namely, the Kwame Nkrumah University Hospital, Kumasi South Hospital and St. Michael’s Hospital. Ethical approval was sought from the Ethics Committee of the hospitals and The Committee on Human Research, Publication and Ethics, School of Medical Sciences, Kwame Nkrumah University of Science and Technology (CHRPE/AP/204/21). Thorough explanation of the study protocol and assurance of anonymity was made to the subjects. Written informed consent was also sought from participants and healthcare management prior to data and samples collection. Participants were first educated on the purpose of the study and only those who gave their consent to participate in the study were recruited. Diagnosis of GDMs was done by a Consultant/Specialist Obstetrician based on the criteria of the International Association of Diabetes and Pregnancy Study Groups (IADPSG), which uses a fasting blood glucose of ≥ 5.1 mmol/L (3). Pregnant women who were already diagnosed of diabetes before pregnancy were excluded.

### Sample Size Justification

The sample size was obtained by the formula:

$\text{n}=\frac{z^{2}p(1-p)}{e^{2}}$ , Where: Z is the standard normal variate at a confidence interval of 95% = 1.96.

p is the prevalence = 10% (10), e is the margin of error = 0.05.


$$\text{n (Minimum number of participants)}=\frac{1.96^{2}(0.10)(1-0.10)}{0.05^{2}}=138$$

Hence a minimum of one hundred and thirty-eighty participants were needed for the study

A 95% confidence level, 50% response distribution, and 5% margin of error at a statistical power of 80% was employed in the calculation of the sample size. To increase statistical power, total of 200 participants were recruited for the study.

### Data Collection

#### Fasting Blood Glucose Measurement

A 3 millilitres of fasting blood samples were collected into fluoride tubes and centrifuged at 3000rpm at 5 minutes. Plasma was analysed for fasting blood glucose (FBG) levels using a fully automated clinical chemistry analyser (LE Scientific, China). Participants who gave their consent had their GDM status confirmed based on the IADPSG criteria using an FBG ≥ 5.1 and were asked to answer a standard questionnaire which provided information on their lifestyle, obstetrics, socio-demographics and clinical conditions.

#### Questionnaire

A well-structured questionnaire was used which had four sections. The first section of the questionnaire was used to assess socio-demographics such as age, ethnicity, Occupational status, family history of diabetes level of education was also, marital status and previous use of oral contraceptives. The second section of the questionnaire assessed the obstetric risk factors associated with gestational diabetes mellitus. This section focused on factors such as history of abortion parity, previous perinatal outcomes, history of caesarean section and gravidity. The third section was used to assess the clinical risk factors of GDM such as obesity, history of hypertension, and history of intrauterine foetal death among others. The fourth section of the questionnaire was used to assess lifestyle risk factors such as physical activity, smoking alcoholism and diet.

### Anthropometric Measurements

Anthropometric measurements such as weight and height were taken to obtain body mass index (BMI) of participants using the weighing scale and stadiometer, respectively. BMI was calculated as weight in kilograms divided by the square of the height in meters. The BMI was classified into 4 categories in accordance with the WHO standard BMI criteria for adults. The categories into; underweight (BMI< 18.5 kg/m2), normal weight (BMI between 18.5 kg/m2 to 24.9 kg/m2), overweight (BMI: 25–29.9 kg/m2), and obese (BMI ≥ 30 kg/m2).

### Statistical Analysis

Collected data were entered in to Microsoft Excel 2016 and analysed using the Statistical Package for Social Sciences (SPSS) Version 23.0 (Chicago IL, USA) and Graph pad prism version 5.0 (Graph Pad software, San Diego California USA, www.graphpad.com).

A descriptive statistic was used to analyse the study variables. Continuous variables were represented by means (± standard deviations) whilst categorical variables by numbers (%).

A bar chart was used to illustrate the prevalence of gestational diabetes among study participants. Univariate logistic regression analysis was performed to screen for potential socio-demographic, obstetric, clinical and lifestyle risk factors associated with gestational diabetes. Multivariate logistic regression modal was used to determine independent risk factors of gestational diabetes.

A p-value of less than 0.05 (*p* < 0.05) and a confidence interval of 95% were chosen as the statistical significance level and confidence interval, respectively.

## Results

### Sociodemographic and Clinical Characteristics of Study Participants

**Table 1** shows the Sociodemographic and clinical characteristics of study participants. A total of 200 participants eligible for the study were included in the final statistical analysis. Of the total participants, one-third (29.5%) were found to be widely distributed between age categories 16 to 25 years followed by 31 to 35 years (28.5%). More than two-fifth of the participants had basic education (44.5%), majority were married (70.0%) and were Akans (69.5%). In addition, two-third of study participants had informal occupation (63.0%) with majority having no family history of hypertension (87.5%) or abortion (75.0%). Moreover, two-third of the participants had no record of previous oral contraceptives use (63.5%), and majority did not have any pregnancy related disorder (95.5%) and had not undergone any caesarean section (83.0%). History on the participant’s previous obstetric risk factors shown that most of the pregnant women previously had termed and vertex delivery, live birth, no macrosomic baby and no previous history of GDM. This corresponds to (65.5%), (65.5%), (93.5%), (81.0%), (96.5%) respectively (**Table 1**).

**Table 1:** Baseline characteristics on socio-demographics and obstetric risk factors.

Variable	Frequency (n = 200)	Percentage (%)
**Age category (Years)**		
16-25	59	29.5
26-30	54	27.0
31-35	57	28.5
36-45	30	15.0
**Educational level**		
No formal education	8	4.0
Basic education	89	44.5
Secondary education	63	31.5
Tertiary education	40	20.0
**Marital status**		
Married	140	70.0
Cohabiting	25	17.5
Single	35	17.5
**Ethnicity**		
Akan	139	69.5
Ewe	3	1.5
Ga-Adangbe	6	3.0
Northerners	52	26.0
**Occupational status**		
Unemployed	33	16.5
Informal	126	63.0
Formal	41	20.5
**Family history of hypertension**		
No	175	87.5
Yes	25	12.5
**History of abortion**		
No	150	75.0
Yes	50	25.0
**Previous use of oral contraceptive**		
No	127	63.5
Yes	73	36.5
**Diagnosis of pregnancy related disorder (s)**		
No	191	95.5
Yes	9	4.5
**Previous caesarean section**		
No	166	83.0
Yes	34	17.0
**Previous delivery status**		
None	54	27.0
Term	131	65.5
Preterm	15	7.5
**Previous presentation at delivery**		
None	60	30.0
Vertex	131	65.5
Breech	9	4.5
**Previous Birth outcomes**		
Live birth	187	93.5
Stillbirth	13	6.5

### Clinical and Lifestyle Factors

**Table 2** shows the clinical and lifestyle characteristics of study participants. Majority of participants neither had preeclampsia (78.5%) nor gestational hypertension (93.0%). Clinically, two-fifth of the participants were overweight (46.2%), majority with no history of intrauterine foetal death (91.0%), threatened abortion (95.5%) or infertility (96.0%). Furthermore, the lifestyle risk factors assessed indicated more than half of the participants eat three times a day (52.5%), take snacks in between meals (56.6%), did not eat late night meals (65.0%) but take in fruits and vegetables regularly (81.5%). In addition, most of the participants exercise regularly (54.0%), and had high preference for carbohydrate food (61.0%) but did not take in neither soda drinks (58.5%) nor fast food (74.0%) (**Table 2**).

**Table 2:** Baseline characteristics on clinical and lifestyle factors.

Variable	Frequency (n = 200)	Percentage (%)
**Previous macrosomic baby**		
No	119	81.0
Yes	28	19.0
**Previous history of Preeclampsia**		
No	157	78.5
Yes	43	21.5
**Previous history of Gestational hypertension**		
No	186	93.0
Yes	14	7.0
**Previous history of GDM**		
No	193	96.5
Yes	7	3.5
**History of intrauterine foetal death**		
No	182	91.0
Yes	18	9.0
**Threatened abortion**		
No	191	95.5
Yes	9	4.5
**History of infertility**		
No	192	96.0
Yes	8	4.0
**Number of daily food intake**		
Once	2	1.0
Twice	29	14.5
Thrice	105	52.5
Four time	45	22.5
More Than Four Times	19	9.5
**Snack in between meals**		
No	87	43.5
Yes	113	56.6
**Late night meals**		
No	130	65.0
Yes	70	35.0
**Regular intake of fruits and vegetables**		
No	37	18.5
Yes	163	81.5
**Regular exercise**		
No	92	46.0
Yes	108	54.0
**Number of exercises per week**		
Daily	82	74.5
Once	4	3.6
Twice	13	11.8
Thrice	5	4.5
More Than Four Times	6	5.5
**Intake of soda drinks**		
No	117	58.5
Yes	83	41.5
**Fast food intake**		
No	148	74.0
Yes	52	26.0
**Preference for carbohydrate food**		
Low	13	6.5
Moderate	65	32.5
High	122	61.0
**BMI status**		
Normal	58	29.1
Overweight	92	46.2
Obese	49	24.6

GDM, Gestational diabetes mellitus; BMI, Body Mass Index.

### Prevalence of Gestational Diabetes Mellitus

**Figure 1:**
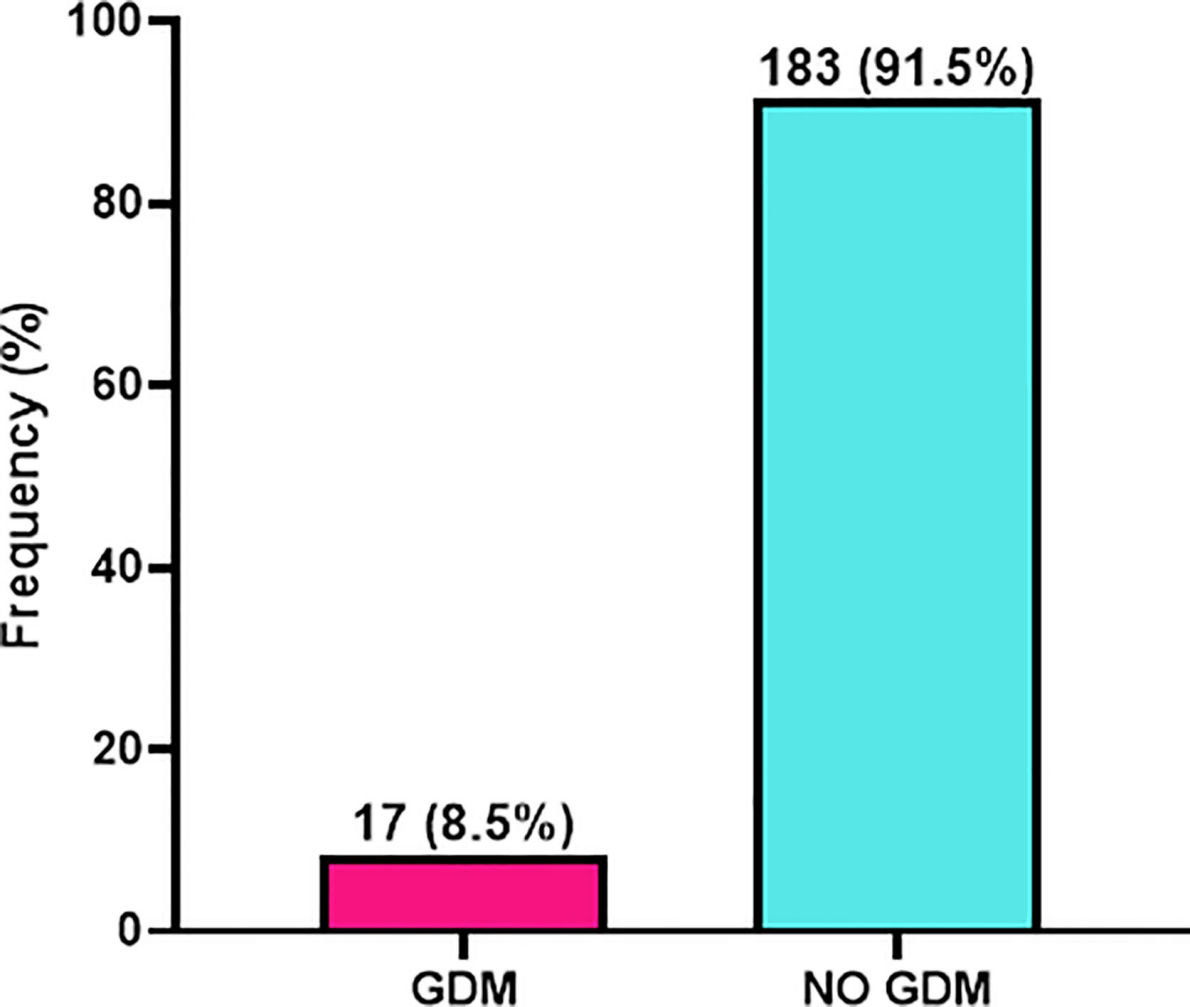
Prevalence of gestational diabetes mellitus (GDM) among study participants.

As shown in **Figure 1**, of the 200 patients eligible for this study, 17 had gestational diabetes which indicated a prevalence of 8.5% whereas 183 (91.5%) were non-GDM patients (**Figure 1**).

### Risk Factors Associated With Gestational Diabetes Mellitus

**Table 3** shows the risk factors associated with gestational diabetes mellitus. In the preliminary univariate analysis, the following study variables had *p*>0.05 and were excluded from the final multivariate logistic regression analysis modal; educational level, ethnicity, occupational status, history of abortion, previous caesarean section, preeclampsia, previous history of gestational diabetes, history of intrauterine foetal death, history of infertility, number of times participants eat in a day, snack in between meals, late night meal, regular exercise, and fast food intake. However, after adjusting for possible confounders in the final multivariate logistic regression model, the use of oral contraceptives [aOR = 13.05, 95%CI = (1.43-119.23), *p* = 0.023] previous history of preeclampsia (aOR: 19.30; 95% CI: 2.15-71.63; p=0.013) and intake of soda drink [aOR =10.05, 95%CI = (1.19-84.73), *p* = 0.034] (**Table 3**).

**Table 3:** Risk factors associated with gestational diabetes.

Variable	cOR (95% CI)	*p*-Value	aOR (95% CI)	*p*-Value
**Age category (Years)**				
16-25	Ref (1)		Ref (1)	
26-30	8.64 (1.03-72.71)	**0.047**	2.96 (0.12-73.33)	0.509
31-35	6.82 (0.80-58.59)	0.080	–	
36-46	6.44 (0.64-64.85)	0.114	–	
**Family history of hypertension**				
No	Ref (1)		Ref (1)	
Yes	3.396 (1.08-10.64)	**0.036**	1.17 (0.10-14.37)	0.903
**Use of oral contraceptive**				
No	Ref (1)		Ref (1)	
Yes	3.58 (1.26-10.13)	**0.016**	13.05 (1.43-119.23)	**0.023**
**Previous history of preeclampsia**				
No	Ref (1)		Ref (1)	
Yes	10.95 (2.62-45.79)	**0.001**	19.30 (2.15-71.62)	**0.013**
**Previous perinatal outcomes**				
None	Ref (1)		Ref (1)	
Live birth	2.17 (0.46-10.26)	0.327	–	
Stillbirth	16.56 (2.74-100.24)	**0.002**	0.006 (0-0.51)	**0.025**
**Previous macrosomic baby**				
No	Ref (1)		Ref (1)	
Yes	4.89 (1.69-14.18)	**0.003**	3.38 (0.37-31.28)	0.283
**Gestational hypertension**				
No	Ref (1)		Ref (1)	
Yes	5.32 (1.47-19.32)	**0.011**	6.15 (0.34-110.06)	0.217
**Threatened abortion**				
No	Ref (1)		Ref (1)	
Yes	18.65 (4.42-78.61)	**0.001**	10.15 (0.53-196.42)	0.125
**BMI**				
Normal	Ref (1)		Ref (1)	
Overweight	2.31 (0.46-11.50)	0.308	–	
Obese	5.46 (1.10-27.09)	**0.038**	8.35 (0.34-205.13)	0.194
**Regular intake of fruits and vegetable**				
No	Ref (1)		Ref (1)	
Yes	0.21 (0.08-0.59)	**0.003**	0.20 (0.02-1.61)	0.13
**Intake of soda drinks**				
No	Ref (1)		Ref (1)	
Yes	7.71 (2.14-27.79)	**0.002**	10.05 (1.19-84.73)	**0.034**

Adjusted for age; cOR; Crude odd ratio, aOR; Adjusted odd ratio, CI; Confidence Interval, Ref; Reference, BMI; Body Mass Index; P-value < 0.05 was considered significant. Bold values indicate significant p-values.

## Discussions

Recently, the incidence of gestational diabetes mellitus (GDM) has increased globally and Africa is not an exception. This study therefore evaluated the prevalence and risk factors of GDM in Kumasi, Ghana. Our study observed a prevalence of 8.5% gestational diabetes mellitus cases among study participants. Previous use of oral contraceptive, and intake of soda drinks as the independent risk factors of gestational diabetes mellitus.

The present study prevalence of 8.5% gestational diabetes is in consistent with that of Anzaku et al. (20), who reported a prevalence of 8.3% among Nigerians (20). On the contrary, the observed prevalence of this study shows a decrease in the prevalence of gestational diabetes compared to that of Oppong et al. (10), who reported 10% prevalence gestational diabetes among Ghanaians. The observed difference in prevalence may be attributed to the present study recruited participants from a clinic setting as opposed to Oppong et al. study conducted at a teaching hospital. Surprisingly, majority of study participants had normal BMI and exercise regularly which explain why we observed a decreased in prevalence of gestational diabetes as compare to previous study reported among Ghanaians.

In this current study, we observed previous use of oral contraceptive as independent risk factor of gestational diabetes. This is in consistent with a study by Kramer et al. (21), who reported the use of oral contraceptives is associated with gestational diabetes (21). The agreement between the current and previous study provides evidence that hormonal contraceptive methods may increase a woman’s risk for GDM since most oral contraceptives are made up of oestrogen and progesterone which in excess induces hypercortisolism and therefore leading to insulin resistance and hyperglycaemia in pregnancy.

Our study observed that, patients with history of preeclampsia were 19-fold more likely to develop GDM. Lee et al. (22), found preeclampsia to be associated with GDM in subsequent pregnancy. This can be explained that the two disease conditions share a common pathophysiology and are characterized by systemic endothelial dysfunction (22).

Another finding of this study was that intake of soda drinks is independently associated with GDM. This finding confirms previous study by Donazar-Ezcurra et al. (23), who reported dietary intake of soda have a strong association with weight gain and metabolic syndrome. This could be explained as leading to spike in insulin which worsen insulin sensitivity overtime thereby enhancing insulin resistance leading to hyperglycaemia.

Previous studies have shown that lifestyle risk factors such as history of smoking, alcoholism and regular exercise are also highly associated with GDM (24). On the contrary, this study did not show similar finding.

Even though the strength of the present study is that it is the first study to be reported in the metropolis, our use of a cross-sectional study design limits this study findings as the casual-effect relationship could not be established. Therefore, it is recommended that a cohort study design should be employed in subsequent studies to help assess more potential risk factors of GDM.

## Conclusion

This study evaluated the prevalence and risk factors associated with GDM in Kumasi, Ghana. We observed GDM prevalence of 8.5% among study participants, which was influenced by previous history of oral contraceptives use, history of preeclampsia and consumption of soda drink

It is recommended that pregnant women be educated on lifestyle modification and the need to reduce consumption of soda drinks and the use oral contraceptives.

## Data Availability Statement

The raw data supporting the conclusions of this article will be made available by the authors, without undue reservation.

## Ethics Statement

The studies involving human participants were reviewed and approved by The Committee on Human Research, Publication and Ethics, School of Medical Sciences, Kwame Nkrumah University of Science and Technology. The patients/participants provided their written informed consent to participate in this study.

## Author Contributions

EOA, WB, and PK. Methodology: EOA, WB, and PK. Formal analysis, PK, ES, and SO. Investigation, WB, EOA, andPK. Original draft preparation, EOA, PK, SO, and ES. Supervision,EOA and WB. All authors listed reviewed, edited have made a substantial, direct, and intellectual contribution to the work and approved it for publication. All authors contributed to the article and approved the submitted version.

## Funding

This study was funded by TiDi Foundation, a non-governmental funding body which supports student research with a seed fund of 140 dollars.

## Conflict of Interest

The authors declare that the research was conducted in the absence of any commercial or financial relationships that could be construed as a potential conflict of interest.

## Publisher’s Note

All claims expressed in this article are solely those of the authors and do not necessarily represent those of their affiliated organizations, or those of the publisher, the editors and the reviewers. Any product that may be evaluated in this article, or claim that may be made by its manufacturer, is not guaranteed or endorsed by the publisher.
